# Effects of Dynamic Core Stability Training as an Adjunct to the FIFA 11+ Program on Dynamic Knee Valgus, Single-Leg Athletic Stability, and Isometric Hip Strength in Amateur Soccer Players: A Randomized Controlled Trial

**DOI:** 10.3390/jfmk11040396

**Published:** 2026-10-01

**Authors:** Muhammad Furqan Hassan, Rabiya Noor, Furqan Ahmed Siddiqi, Muhammad Salman Bashir

**Affiliations:** 1 Department of Physical Therapy, Riphah International University, Lahore 54660, Pakistan; 2 Foundation University Islamabad, Islamabad 44000, Pakistan; furqan@fui.edu.pk; 3 School of Health Sciences, University of Management & Technology, Lahore 54770, Pakistan; salman.bashir@umt.edu.pk

**Keywords:** core stability, dynamic knee valgus, FIFA 11+, single-leg athletic stability, soccer

## Abstract

**Background and Objectives**: Proximal trunk and hip control during dynamic tasks is a key modifiable determinant of dynamic knee valgus (DKV) in soccer players. This study investigated the effects of adding dynamic core stability training (DCS) to the FIFA 11+ warm-up program compared with the FIFA 11+ on DKV, single-leg athletic stability, and isometric hip muscle strength in amateur soccer players. **Methods:** A single-blinded, parallel-group randomized controlled trial was conducted on 40 amateur soccer players (mean age 16.6 ± 3.1 years). Participants were randomly allocated to either DCS+FIFA 11+ training or FIFA 11+ alone, with both interventions performed three times per week for 8 weeks. DKV, single-leg athletic stability and isometric hip strength was assessed at the baseline, 4 weeks, and 8 weeks. Data were analyzed using linear mixed-effects models to examine Group, Time, and Group × Time effects, with participant included as a random effect. Benjamini–Hochberg false discovery rate adjustment was applied to the Group × Time comparisons. **Results:** Significant Time effects were observed for all outcomes. After Benjamini–Hochberg correction, significant Group × Time interactions were observed for left-leg athletic stability (adjusted *p* < 0.001; ηp^2^ = 0.243), left hip abductor strength (adjusted *p* = 0.025; ηp^2^ = 0.125), and right and left hip external rotator strength (adjusted *p* < 0.001 and <0.001; ηp^2^ = 0.302 and 0.404 respectively), with greater improvements in the DCS+FIFA 11+ group. Both groups demonstrated reductions in right and left DKV over time; however, Group × Time interactions were not significant for either DKV measure. **Conclusions:** Adding dynamic core stability training to the FIFA 11+ was associated with greater longitudinal improvements in left-leg athletic stability and hip muscle strength outcomes, particularly left hip abductor and bilateral hip external rotator strength. Supplementing the FIFA 11+ with DCS training may provide additional benefits for selected aspects of lower-extremity movement control and proximal muscle strength in amateur soccer players.

## 1. Introduction

Soccer is one of the most popular and widely played sports worldwide and places substantial physical demands on players [1]. The lower extremity is frequently affected, with injuries to the thigh, knee, and ankle accounting for approximately 60–90% of reported soccer-related injuries [2]. Amateur soccer players are regularly exposed to repeated running, acceleration, deceleration, and other sport-specific movement demands during training and competitive play [3]. Moreover, soccer involves frequent unilateral and multi-planar movements, including cutting, kicking, landing, and rapid changes in direction, which require adequate muscle strength, single-leg stability, postural control, and core stability. Deficits in any of these neuromuscular components may compromise lower-extremity movement control during sport-specific activities [4,5,6,7]. Hence, maintaining effective neuromuscular control and coordinated movement is an important consideration in amateur soccer players.

Effective core activation is particularly essential during single-leg stance, cutting, landing, and deceleration, where proximal stability facilitates optimal lower-extremity control and movement efficiency [8,9]. Inadequate core stability may alter lower extremity biomechanics, potentially contributing to increased dynamic knee valgus (DKV) [10]. DKV is a multi-planar movement pattern characterized by excessive medial knee displacement as a result of hip internal rotation and adduction, altered tibial rotation, and impaired ankle mechanics during weight-bearing activities such as squatting, jumping, landing, and change in direction [11]. DKV has been widely described and investigated in relation to ACL injury mechanisms [11,12]. The hip and knee muscles play a central role in regulating knee alignment in the frontal and transverse planes. Reduced gluteal and hamstring strength or activation has been associated with greater knee valgus during landing [13]. Consistent with this, warm-up protocols targeting hip external rotators and abductors have been shown to significantly reduce DKV in youth male soccer players [14].

To address these challenges, the Fédération Internationale de Football Association (FIFA) developed a structured warm-up program—the FIFA 11+, comprising running, strength, balance, and plyometric exercises [15]. The program has been associated with reductions in overall and lower-extremity injury incidence [16] and improvements in neuromuscular characteristics such as hamstring strength and balance in soccer players [17]. A six-week study comparing the FIFA 11+ with a new warm-up program (NWP) reported greater improvements in balance and muscle strength following the NWP, suggesting that different warm-up exercise configurations may produce different neuromuscular adaptations [18].

Given the role of trunk control in lower-extremity biomechanics during soccer-specific movements, dynamic core stability (DCS) training represents a potential approach for targeting proximal control [19]. Although the FIFA 11+ program incorporates exercises intended to improve neuromuscular control and also includes core stability exercises, it contains relatively more static than dynamic core activities [20]. Dynamic core exercises may impose greater demands on trunk control, proprioception, muscular activation, and joint stabilization [20]. Supporting the potential value of supplementary exercises, previous studies have demonstrated that the addition of core stability, eccentric, and proprioceptive exercises to regular soccer training can improve balance, lower extremity strength, and core muscle performance [21]. Furthermore, a six-week DCS program in male soccer players has been shown to alter selected lower-limb biomechanical variables, including greater hip extensor moments and reduced internal knee valgus moments [19]. However, these findings do not establish whether incorporating DCS exercises into an established warm-up program such as the FIFA 11+ provides additional benefits beyond the FIFA 11+ alone. Specifically, limited evidence is available regarding whether the addition of DCS to FIFA 11+ results in greater improvements in DKV, single-leg athletic stability, and isometric hip strength in amateur soccer players. Addressing this question is relevant because these outcomes reflect proximal lower-extremity control and neuromuscular characteristics involved in soccer-specific movement.

Therefore, this study investigated the effects of adding dynamic core stability training to the FIFA 11+ program on dynamic knee valgus, single-leg athletic stability, and isometric hip strength in amateur soccer players. The primary outcomes were dynamic knee valgus and single-leg athletic stability, while hip muscle strength measures were secondary outcomes. We hypothesized that participants who received DCS training in addition to FIFA 11+ would demonstrate greater improvements in dynamic knee valgus, single-leg athletic stability, and isometric hip strength compared with those who received the FIFA 11+ program alone.

## 2. Materials and Methods

A two-arm, parallel-group, randomized controlled trial was conducted at POPO Football Club and Foundation University College of Physical Therapy, Foundation University Islamabad, between October 2022 and April 2024. Participants were enrolled in separate cohorts rather than simultaneously. This was carried out to accommodate participant availability and to allow the supervised intervention sessions and outcome assessments to be conducted appropriately within the study settings. Each cohort completed the same standardized 8-week intervention and assessment. The coaching environment for the study intervention was therefore consistent across cohorts. This was a single-blinded study, and the outcome assessor was blinded to group allocation.

### 2.1. Participants

**Figure 1:**
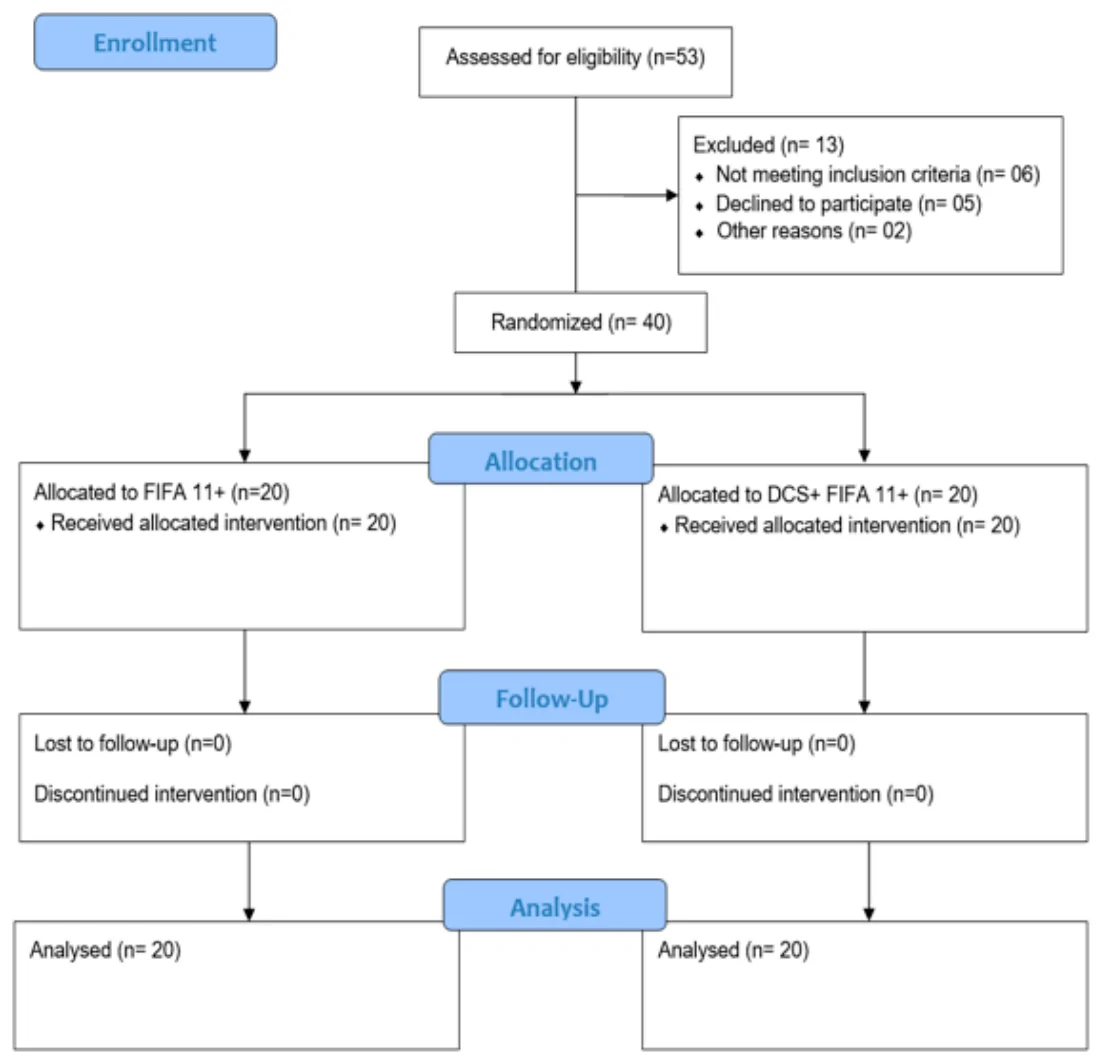
CONSORT diagram illustrating participant recruitment, allocation, follow-up & analysis.

An a priori sample-size calculation was performed using G*Power version 3.1.9.4 based on data from the pilot study, with DKV as the primary outcome. Using a two-tailed independent-samples *t*-test, an α level of 0.05, 80% statistical power (1 − β = 0.80), an allocation ratio of 1:1, and an estimated Cohen’s d of 0.91 derived from the pilot data, the sample size was calculated as 40 participants (20 per group). The original calculation was based on an independent-group comparison and did not incorporate assumptions regarding within-participant correlation or sphericity for repeated measurements. No additional allowance for attrition was applied. All 40 randomized participants completed the study and all scheduled assessments. Participants were recruited through non-probability convenience sampling and randomly allocated to one of the two interventional groups (Figure 1). Eligible participants were male or female soccer players aged 13–25 years who trained regularly at the selected football academies. Exclusion criteria included a history of musculoskeletal disorders or lower-extremity fractures. Participants were excluded if they had sustained a recent injury resulting in 10–30 consecutive days of absence from full training or match participation. This criterion was used to exclude players with a recent time-loss injury that could potentially influence baseline physical performance and movement characteristics and is consistent with established football injury-severity classifications [22]. Individuals using nutritional supplements were also excluded to minimize supplementation as a potential source of variability in physical performance and training adaptations. Participants were not involved in the design and reporting of the trial; however, coaches facilitated the recruitment process by identifying and informing eligible players about the study.

### 2.2. Ethical Considerations

Ethical approval was obtained from the Review and Ethics Committee of Riphah College of Rehabilitation and Allied Health Sciences (REC/RCR & AHS/21/1105), and all the study procedures followed the principles of the Declaration of Helsinki. The trial was also registered with ClinicalTrials.gov (NCT04958837). Before enrollment in the study, written informed consent was taken from all participants aged 18 years or older. For participants younger than 18 years, written informed consent was obtained from their parent or legal guardian, and written assent was obtained from the participant. Participants were informed that they could withdraw from the study at any time without any consequences. Participant confidentiality was preserved throughout the study. Trial reporting followed the Consolidated Standards of Reporting Trials (CONSORT) guidelines.

### 2.3. Training Interventions

Study subjects were allocated in a 1:1 ratio to either the FIFA 11+ group or the DCS+FIFA 11+ group, using a computer-generated simple randomization sequence. Randomization was not stratified according to age or sex. To ensure allocation concealment, an independent researcher with no involvement in the study prepared opaque, sequentially numbered, sealed envelopes containing the group assignments. The primary researchers opened the envelopes sequentially after the baseline assessments and assigned participants to their respective groups. The randomization sequence was also generated by the same independent researcher who prepared the sealed envelopes.

Participants in the FIFA 11+ group completed the standard FIFA 11+ program, including its prescribed progression criteria [15]. For progression, participants advanced to the subsequent difficulty level according to the standard FIFA 11+ protocol as given in Table 1. The DCS+FIFA 11+ group completed the same FIFA 11+ program followed by additional dynamic core stability exercises. For the DCS component, the progression was standardized across the intervention period. Exercises were advanced during weeks 5–8 by increasing the volume of the repetitive dynamic exercises while maintaining a constant 60-s inter-set recovery, based on the general principle of progressive increases in training volume for muscle endurance development [23]. The supine bridge was progressed from bilateral to unilateral support to increase the lumbopelvic stability and control demand. These exercises were classified as dynamic core stability exercises because they required active trunk and lumbopelvic control during dynamic limb or body-segment movements, consistent with previous research describing dynamic core stability training [24]. The detailed protocol provided to the DCS+FIFA 11+ group is presented in Table 2.

The dynamic core stability component required approximately 20–25 min per session (including rest breaks). Thus, the total duration of each training session was approximately 20 min in the FIFA 11+ group and 35–40 min in the DCS+FIFA 11+ group. All training sessions were conducted under direct supervision, three times per week on alternate days, for 8 weeks. Supervision was carried out to ensure correct exercise execution and adherence to the prescribed protocol. Participant adherence was monitored using an attendance log documenting the number of sessions attended by each participant. The planned intervention consisted of 24 sessions over 8 weeks in both groups. Participants continued their usual soccer-specific training during the study period; however, no soccer matches were played during this period.

### 2.4. Outcome Measures

The outcomes included DKV, single-leg athletic stability, and isometric hip muscle strength. All assessments were conducted under standardized conditions at the baseline, 4 weeks, and 8 weeks.

#### 2.4.1. Dynamic Knee Valgus Assessment

DKV was assessed using the MotionMetrix markerless gait analysis system (MotionMetrix AB, Lidingö, Sweden) integrated with Microsoft Kinect™ sensors (Version 1.0, Microsoft Corporation, Redmond, WA, USA). The system has demonstrated test–retest reliability for several walking and running gait parameters [25], and has shown agreement with an optoelectronic 3-D motion capture system for gait and lower-extremity kinematic measurements [26]. The treadmill was positioned within the calibrated capture area of the MotionMetrix system. Two Kinect sensors were positioned on either side of the treadmill, approximately 170 cm anterior to the treadmill and 190 cm lateral to it, and calibrated before each assessment. Before testing, the participants’ anthropometric characteristics required for the MotionMetrix analysis were entered into the system. Following familiarization, participants ran on the treadmill for 30 s at a self-selected comfortable speed. Treadmill running was selected as a standardized and repeatable locomotor task for assessing frontal plane knee alignment under controlled conditions. This approach enabled consistent assessment across time points and reduced variability in task execution. The speed selected at the baseline assessment was recorded and maintained for each participant during all subsequent assessment sessions. DKV was operationalized using the MotionMetrix “Knee alignment @ mid-stance” variable, representing the frontal-plane knee alignment angle in degrees, at mid-stance during running. The outcome was recorded separately for the right and left limbs. Positive values represented valgus alignment, whereas negative values represented varus alignment. All measurements were performed using the same equipment, spatial configuration, calibration procedure, and testing protocol for all participants.

#### 2.4.2. Single-Leg Athletic Stability Assessment

Single-leg athletic stability for each limb was assessed by the Biodex Balance System SD (Biodex Medical Systems, Shirley, NY, USA) according to standardized testing procedures and quantified using the Overall Stability Index (OSI). Participants were tested separately for each limb while standing barefoot on the dynamic platform. For each trial, the tested foot was positioned on the platform, and the heel position and foot angle were recorded using the Biodex system’s platform grid to ensure consistent placement across trials. The Athlete Single Leg Stability Test was conducted with the eyes open, with platform stability levels progressively set from an initial level of 8 (more stable) to an ending level of 3 (less stable). Participants maintained an upright single-leg stance while focusing on the visual feedback displayed on the monitor and attempting to keep the platform as stable as possible. One practice trial was completed for familiarization, followed by three 20-s trials for each limb. A 10-s rest period was provided between consecutive tests. The mean score of the three trials was calculated for each limb, with lower scores indicating better stability [27,28].

#### 2.4.3. Isometric Hip Muscle Strength Assessment

Hip muscle strength was assessed by measuring maximal voluntary isometric contraction (MVIC) using a hand-held dynamometer (microFET^®^2, Hoggan Scientific, LLC, Salt Lake City, UT, USA). The assessment protocol was based on previously described procedures for hand-held dynamometry [29]. All strength assessments were performed by the same trained examiner using a standardized testing procedure. Participants were assessed in three standard positions (sitting, supine, and prone) to minimize repositioning and improve the practicality of the assessment. Hip flexors were assessed in the sitting position, hip abductors and adductors in the supine position, and hip extensors in the prone position. All tests involved maximal voluntary isometric contractions.

For hip flexor strength, participants were seated with the hip and knee flexed to 90°. The dynamometer was positioned against the anterior aspect of the distal thigh, just proximal to the knee joint. For hip extensor strength, participants were positioned prone with the hip and knee extended, and the dynamometer was placed against the posterior aspect of the distal thigh, just proximal to the knee joint. For hip abductor strength, participants were positioned supine with the hip and knee extended, and the dynamometer was placed against the lateral aspect of the distal limb. For hip adductor strength, the same position was used, with the dynamometer positioned against the medial aspect of the distal limb. The examiner provided manual stabilization and held the dynamometer in a fixed position to minimize compensatory movements during each contraction [29].

Hip internal and external rotator strength was assessed with the participant seated at the edge of the couch, and the hip and knee flexed to approximately 90°. The trunk and pelvis were stabilized to minimize compensatory movements. For hip internal rotation, the dynamometer was positioned against the medial aspect of the distal lower leg, just proximal to the medial malleolus, with resistance applied in the direction of external rotation. For hip external rotation, the dynamometer was positioned against the lateral aspect of the distal lower leg, just proximal to the lateral malleolus, with resistance applied in the direction of internal rotation. Participants were instructed to produce a maximal voluntary isometric contraction against the dynamometer while maintaining the standardized testing position [30].

Before formal testing, one submaximal practice trial was provided for each muscle group to familiarize participants with the procedure and ensure proper execution of the contraction. Participants were then instructed to produce a maximal isometric contraction against the dynamometer following a standardized verbal command. Each maximal contraction was maintained for approximately 3–5 s, consistent with the previously described protocol [29]. Strength values were recorded as force in Newtons (N) and were not normalized to body mass. Two maximal trials were recorded for each muscle group, with a 60-s rest interval between trials to minimize fatigue and allow adequate recovery. A trial was considered acceptable when the participant maintained the prescribed testing position and produced a sustained contraction without observable compensatory movement or loss of positioning. The highest value obtained from the two acceptable trials was used for statistical analysis.

### 2.5. Statistical Analysis

Statistical analyses were conducted through IBM SPSS Statistics, version 26.0 (IBM Corp., Armonk, NY, USA). Continuous variables were reported as mean ± standard deviation (SD), while categorical variables were summarized as frequencies and percentages. Longitudinal changes in DKV, single-leg athletic stability, and isometric hip muscle strength were examined using linear mixed-effects models (LMMs). A two-sided significance level of α = 0.05 was used. For each outcome, Group (DCS+FIFA 11+ vs. FIFA 11+), Time (baseline, 4 weeks, and 8 weeks), and the Group × Time interaction were specified as fixed effects, with participant identification code included as the random-effects grouping factor to account for the correlation of repeated observations within participants. Models were estimated using restricted maximum likelihood (REML), with Satterthwaite approximation used for denominator degrees of freedom. With three assessment points, the Group effect had 1 numerator degree of freedom, whereas the Time and Group × Time effects each had 2 numerator degrees of freedom. The corresponding denominator degrees of freedom were 38 for the Group effect and 76 for the Time and Group × Time effects.

The Group × Time interaction was considered the principal test of whether longitudinal changes differed between the intervention groups. Estimated marginal means (EMMs) were calculated for each group at the baseline, 4 weeks, and 8 weeks and are reported with 95% confidence intervals. Partial eta squared (ηp^2^) was used to quantify the magnitude of the Group × Time interaction effects. Bonferroni adjustment was retained for within-group pairwise comparisons. Since 16 Group × Time interaction tests were performed across the longitudinal outcomes, Benjamini–Hochberg false discovery rate (FDR) adjustment was applied to control for multiple comparisons. Both nominal and FDR-adjusted *p*-values were reported and considered when interpreting differential longitudinal effects. FDR-adjusted *p*-values were used to determine statistical significance of the Group × Time interactions. All 40 participants completed the assessments at the baseline, 4 weeks, and 8 weeks; therefore all 120 observations were available for the longitudinal analysis and no missing-data imputation was required.

## 3. Results

### 3.1. Participants Characteristics & Adherence

Forty amateur soccer players were randomized and allocated equally to the two intervention groups. Participants in the DCS + FIFA 11+ group attended 23.65 ± 0.61 of the 24 planned sessions, corresponding to an adherence rate of 98.54 ± 2.54%, whereas participants in the FIFA 11+ group attended 23.80 ± 0.52 sessions, corresponding to 99.17 ± 2.17% adherence. No formal minimum attendance threshold was pre-specified. Nevertheless, adherence was high in both groups, as reported above. There were no major protocol deviations recorded in each group. All participants completed the training and follow-up assessments, with no dropouts or adverse events reported during the intervention period. Data from all of the participants who were randomized were included in the final analysis. Right leg dominance was observed in 80% of the participants. Baseline demographic and anthropometric characteristics of the participants are presented in Table 3. No statistically significant between-group differences were observed for the assessed baseline characteristics (all *p* > 0.05).

### 3.2. Linear Mixed Model Analysis

Longitudinal changes in the outcomes were examined using linear mixed-effects models including Group, Time, and Group × Time as fixed effects, with participant identification code as the random-effects grouping factor. Overall, Time had statistically significant effects for all outcomes (all *p* ≤ 0.009), indicating changes across the three assessment points. However, before adjustment for multiple comparisons, significant Group × Time interactions were observed for left-leg athletic stability, left hip flexors, left hip abductors, left hip adductors, and bilateral hip external rotators strength. No statistically significant Group × Time interaction was observed for either DKV measure or right-leg athletic stability.

To account for multiple Group × Time comparisons across the 16 longitudinal outcome tests, the Benjamini–Hochberg FDR procedure was applied. After FDR adjustment, significant interactions remained for left-leg athletic stability (adjusted *p* <0.001), left hip abductors (adjusted *p* = 0.025), and right and left hip external rotator strength (adjusted *p* <0.001 and <0.001, respectively). The interactions for left hip flexors and left hip adductor strength were not significant after FDR adjustment (adjusted *p* = 0.119 and 0.119, respectively) (Table 4).

### 3.3. Dynamic Knee Valgus

**Figure 2:**
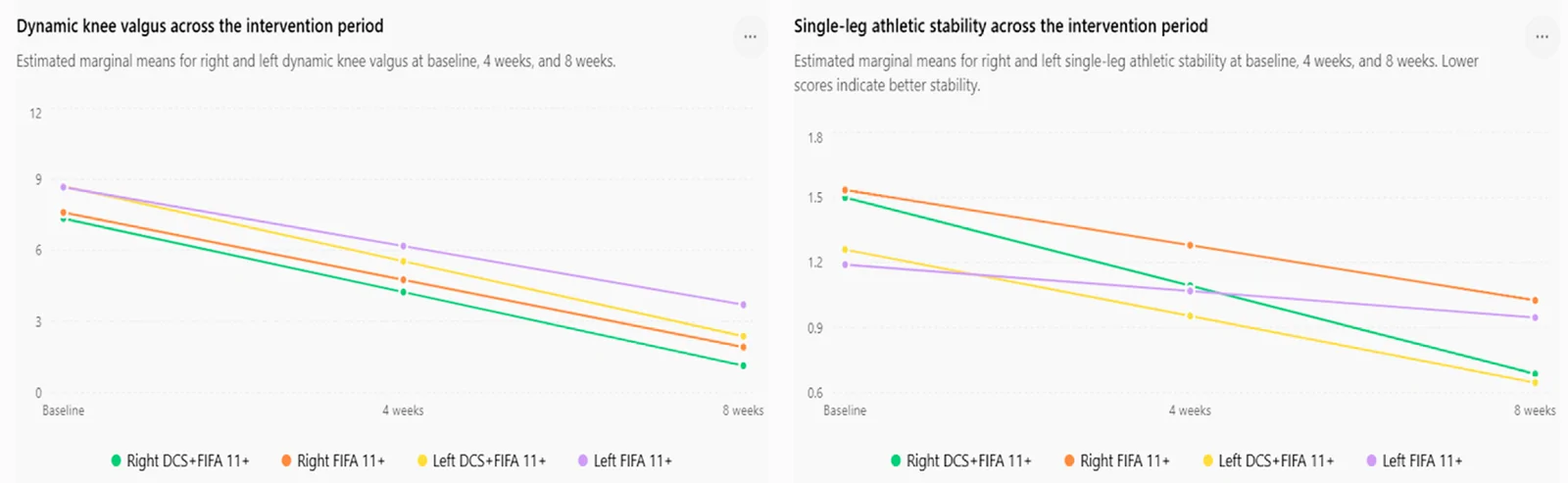
Estimated marginal means of DKV and single-leg athletic stability.

For right DKV, there was a significant effect of Time, F(2,76) = 285.243, *p* < 0.001, but neither the main effect of Group, F(1,38) = 2.243, *p* = 0.142, nor the Group × Time interaction, F(2,76) = 0.565, *p* = 0.571, was statistically significant. Estimated marginal means decreased from 7.35° at the baseline to 4.24° at 4 weeks and 1.13° at 8 weeks in the DCS+FIFA 11+ group compared with 7.60°, 4.76°, and 1.91°, respectively, in the FIFA 11+ group (Figure 2).

For left DKV, Time also had a significant effect, F(2,76) = 195.882, *p* < 0.001. The main effect of Group was not significant, F(1,38) = 3.368, *p* = 0.074, and the Group × Time interaction did not reach statistical significance, F(2,76) = 2.874, *p* = 0.063. Estimated marginal means decreased from 8.71° to 2.37° in the DCS+FIFA 11+ group and from 8.67° to 3.70° in the FIFA 11+ group over the 8 weeks. Thus, both groups demonstrated a reduction in DKV over time, but the longitudinal difference between groups was not statistically significant. (Table 5).

### 3.4. Single-Leg Athletic Stability

For right-leg athletic stability, a significant effect of Time was observed, F(2,76) = 53.527, *p* < 0.001, whereas the main effect of Group was not significant, F(1,38) = 1.237, *p* = 0.273. The Group × Time interaction was also not statistically significant, F(2,76) = 2.836, *p* = 0.065. Estimated marginal means decreased from 1.50 at the baseline to 0.69 at 8 weeks in the DCS+FIFA 11+ group and from 1.54 to 1.03 in the FIFA 11+ group. Because lower scores represent better stability, these findings indicate improvement over time in both groups, without a statistically significant differential longitudinal effect between interventions (Figure 2).

For left-leg athletic stability, significant effects were observed for Time, F(2,76) = 65.742, *p* < 0.001, and Group × Time, F(2,76) = 12.169, *p* < 0.001. The main effect of Group was not significant, F(1,38) = 0.673, *p* = 0.417. Estimated marginal means decreased from 1.26 to 0.65 in the DCS+FIFA 11+ group and from 1.19 to 0.95 in the FIFA 11+ group over the 8-week intervention, with the significant interaction indicating different longitudinal trajectories between groups (Table 5).

### 3.5. Isometric Hip Muscle Strength

Significant main effects of Time were observed for all hip strength outcomes (all *p* ≤ 0.009), indicating changes across the three assessment points. After Benjamini–Hochberg FDR adjustment, statistically significant Group × Time interactions were observed for left hip abductors (F(2,76) = 5.423, nominal *p* = 0.006, adjusted *p* = 0.025) and right and left hip external rotator strength (F(2,76) = 16.427 and 25.752, respectively; both nominal and adjusted *p* < 0.001). For these outcomes, estimated marginal means indicated greater increases over the 8-week intervention in the DCS+FIFA 11+ group than in the FIFA 11+ group (Table 5).

**Figure 3:**
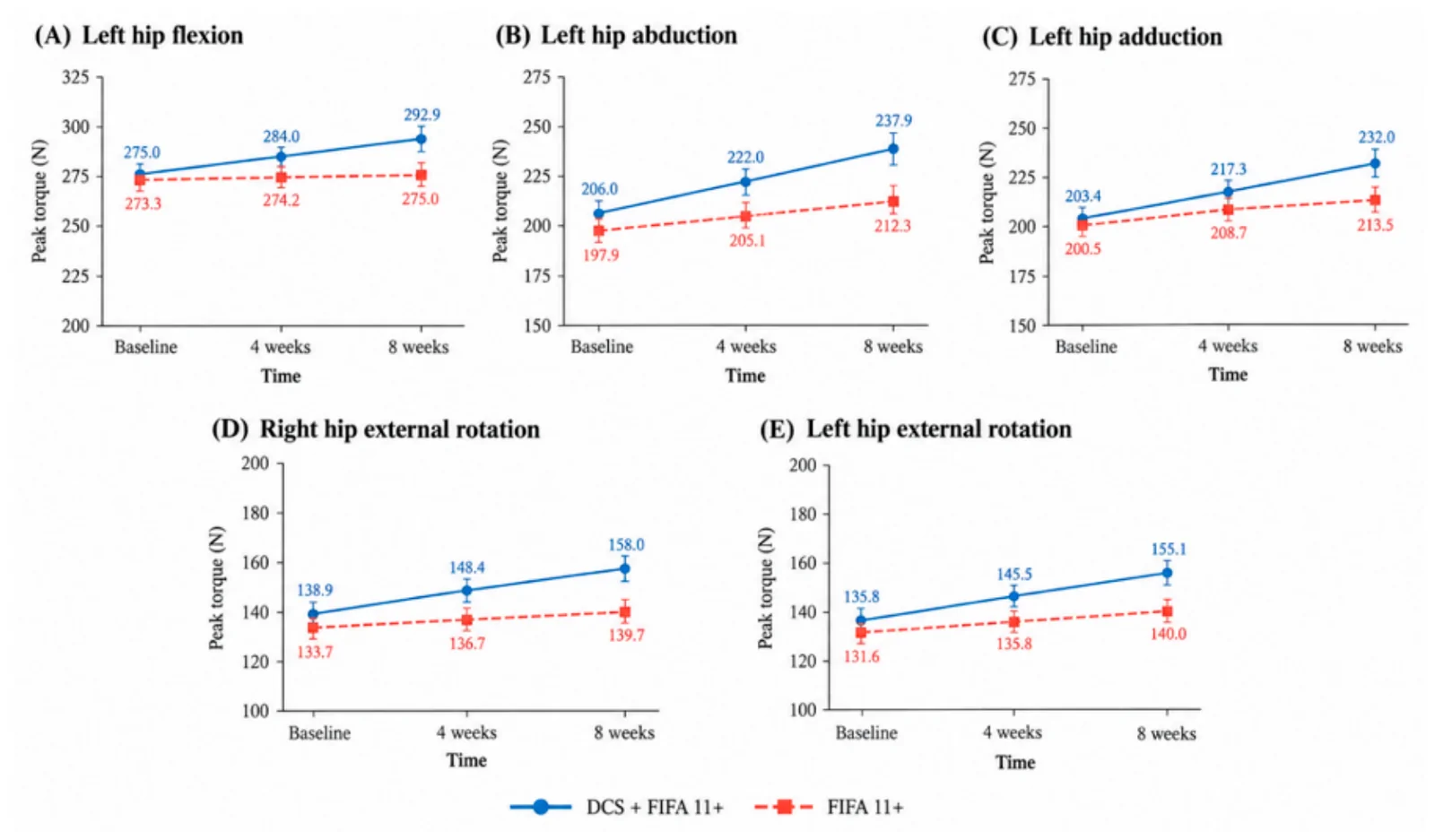
Estimated marginal means of hip strength outcomes with significant Group × Time interactions. Estimated marginal means (± standard errors) for (**A**) left hip flexion, (**B**) left hip abduction, (**C**) left hip adduction, (**D**) right hip external rotation, and (**E**) left hip external rotation at baseline, 4 weeks and 8 weeks. Values are derived from the linear mixed-effects models.

Nominally significant but FDR-adjusted non-significant Group × Time interactions were observed for left hip flexors (F(2,76) = 3.435, nominal *p* = 0.037, adjusted *p* = 0.119) and left hip adductors (F(2,76) = 3.238, nominal *p* = 0.045, adjusted *p* = 0.119). These findings were therefore not considered statistically significant after adjustment for multiple comparisons. No significant Group × Time interactions were observed for bilateral hip extensors, right hip flexors, right hip abductors, right hip adductors, or bilateral hip internal rotators (all adjusted *p* > 0.05) (Figure 3).

## 4. Discussion

This study compared the effects of supplementing the FIFA 11+ program with dynamic core stability training (DCS+FIFA 11+) versus FIFA 11+ alone in amateur soccer players. Specifically, the present study evaluated biomechanical outcomes, as well as stability and strength measures. Both interventions were associated with improvements across the assessed outcomes. However, after adjustment for multiple comparisons, the addition of DCS resulted in significantly greater longitudinal improvements in left-leg athletic stability, left hip abductor strength, and bilateral hip external rotator strength compared with FIFA 11+ alone.

The FIFA 11+ warm-up program itself has been shown to improve several biomechanical measures, core stability, and balance following mid-to-long-term implementation [20]. In the present study, dynamic knee valgus decreased progressively in both groups over the 8-week intervention period. Although the numerical reduction was greater in the DCS+FIFA 11+ group for both limbs, the Group × Time interactions were not statistically significant, indicating that the addition of DCS did not result in a statistically greater reduction in DKV compared with FIFA 11+ alone. This finding is consistent with Seyedi et al., who reported reduced knee valgus following an 8-week FIFA 11+ program in adolescent soccer players [31]. Findings from a study by Whyte et al. also demonstrated favorable changes in knee valgus angles and external hip rotator moments following a dedicated core stability training program [19]. Collectively, these findings suggest that both the FIFA 11+ and targeted dynamic core training may be associated with improvements in lower-extremity movement mechanics; however, the present results do not provide evidence of an additional effect of incorporating DCS on DKV beyond FIFA 11+ alone.

In contrast, a 6-week strengthening program did not significantly improve DKV in young football players [32]. Differences in intervention duration, exercise selection, and training characteristics may partly explain these contrasting findings. The potential contribution of proximal stabilization is also supported by Ambrus et al., who reported improvements in knee valgus following a pelvic stabilization exercise program in individuals with DKV [33]. More broadly, proximal and lower-extremity neuromuscular control have been identified as modifiable factors associated with DKV, highlighting the relevance of exercise-based strategies targeting these components [34].

In the present study, a significant Group × Time interaction was observed for left-leg single-leg athletic stability, indicating greater longitudinal improvement with DCS+ FIFA 11+ compared with FIFA 11+ alone. Single-leg activities place considerable demands on coordinated activation of the trunk and hip musculature to maintain postural control [35]. The dynamic and functional nature of the additional DCS exercises may have provided an additional stimulus for coordinated trunk and lower-extremity control [36]. These findings are in line with a recent systematic review and meta-analysis reporting improvements in balance following core stability training in soccer players [37]. Improvements in balance have also been reported following 8 weeks of FIFA 11+ training in youth football players [38]. Thus, the improvement observed in both groups in the current study may partly reflect the balance and neuromuscular components already incorporated within the FIFA 11+ program, while the significant Group × Time interaction suggests an additional contribution associated with DCS training.

The side-specific nature of this finding warrants consideration. Although a significant Group × Time interaction was observed for left-leg athletic stability, the corresponding interaction for the right leg was not significant. Most participants in the present study were right-foot dominant, and the left non-dominant limb may have been more frequently used as the support or stance limb during soccer actions such as kicking. This difference in functional role may have contributed to the differing responses between limbs. Consistent with the present findings, Dafkou et al. reported greater improvements in hamstring muscle strength and single-leg balance of the non-dominant limb following a supplementary program incorporating postural balance, core, and eccentric exercises in soccer players [21]. In contrast, a study on young soccer players using the Balance Error Scoring System (BESS) did not demonstrate additional improvements in postural control following core training [39]. This discrepancy may reflect differences in intervention characteristics and outcome assessment methods. The Biodex Balance System used in the present study provides quantitative, instrumented measures of postural stability, whereas BESS relies on the scoring of observable balance errors.

The DCS+FIFA 11+ group demonstrated greater longitudinal improvements in selected measures of isometric hip muscle strength. After adjustment for multiple comparisons, significant Group × Time interactions were observed for left hip abductor strength and bilateral hip external rotator strength. These findings are broadly consistent with previous research reporting improved neuromuscular function after core stabilization training in athletic populations [40]. Similarly, Prieske et al. demonstrated neuromuscular adaptations in youth soccer players after core-focused training [41]. In the present study, the DCS training component involved dynamic, multiplanar exercises requiring coordinated trunk and lower-extremity control. Such exercises may provide a stimulus for integrated neuromuscular function involving the hip and trunk musculature [36], which may partly account for the observed improvements in selected hip strength measures.

The findings for hip abductor strength also showed a side-specific pattern. A significant Group × Time interaction was observed for the left hip abductors, whereas the corresponding effect was not observed on the right side, similar to the findings of single-leg athletic stability. This is consistent with previous evidence linking hip muscle function with lower-extremity alignment during single-leg tasks [34]. Likewise, significant longitudinal interactions were observed for hip external rotator strength bilaterally, indicating that the pattern of response was not uniform across all hip muscle groups. Differences in task demands or limb-specific adaptations may contribute to this variation.

Overall, both training approaches produced improvements across the assessed outcomes, while the addition of DCS exercises was associated with greater longitudinal improvements in selected measures of single-leg stability and hip muscle strength. These findings may have practical implications for coaches and physiotherapists working with amateur soccer players, particularly when proximal control, single-leg stability and hip muscle strength are targeted. However, the additional training time required for DCS exercises should also be considered when integrating them into existing FIFA 11+ routines.

This study has several methodological strengths. The randomized controlled design, concealed allocation, and blinded outcome assessment reduced the potential for selection and assessment bias. Moreover, the study assessed several clinically relevant outcomes for amateur soccer players using standardized and objective assessment tools. Assessments at three time points also enabled changes in these outcomes to be examined across the intervention period.

Nevertheless, the study had several limitations. First, the sample size was comparatively small and no female participants were recruited during the study period, thus limiting the generalizability of the findings across sex. Moreover, the relatively broad age range of the participants encompassed both adolescent and young adult soccer players. Age- and maturation-related differences may influence neuromuscular control, muscle strength, and knee biomechanics, and may therefore contribute to variability in the observed responses. Future studies should include female players and consider narrower or maturation-stratified age ranges.

Second, the DCS+FIFA 11+ group received a greater overall training exposure than those in the FIFA 11+ group because of the additional DCS component. Therefore, although greater longitudinal improvements were observed for selected outcomes in the combined training group, the independent contribution of the dynamic core component cannot be completely separated from the potential effect of the greater total training dose. Third, hip strength was reported as absolute force (N) rather than normalized values, which may limit a direct comparison of strength values among participants with different body sizes. Future studies should consider appropriate normalization procedures when comparing muscle strength across heterogeneous samples.

Finally, the assessments were conducted under controlled testing conditions and may not fully reflect the demands of competitive match play. Further research should incorporate sport-specific assessments and longer-term follow-up to examine the transfer and retention of training effects.

## 5. Conclusions

In conclusion, the addition of dynamic core stability training to the FIFA 11+ program was associated with greater improvements in left-leg athletic stability and isometric hip muscle strength, specifically, hip abductor and bilateral hip external rotator strength in amateur soccer players. However, the reduction in dynamic knee valgus did not differ significantly between the two training approaches. The addition of DCS may therefore offer a useful approach for achieving additional improvements in selected aspects of lower-extremity movement control and hip muscle strength beyond the effects of FIFA 11+ alone.

## Figures and Tables

**Table 1:** Detailed protocol of the FIFA 11+ warm-up program.

**Part 1. Running Exercises (Approx. 8 min)**		
**Exercise**		**Repetitions**
Running straight ahead		2
Running hip out		2
Running hip in		2
Running circling partner		2
Running shoulder contact		2
Running quick forward and backward		2
**Part 2. Strength, Plyometrics, and Balance (Approx. 10 min)**		
**Exercise**	**Level**	**Repetitions/Duration**
2A. The Bench	Level 1: Static	3 reps × 20–30 s
	Level 2: Alternate leg	3 reps × 20–30 s
	Level 3: One-leg lift and hold	3 reps × 20–30 s
2B. Sideways Bench	Level 1: Static	3 reps × 20–30 s (each side)
	Level 2: Raise and lower hip	3 reps × 20–30 s (each side)
	Level 3: With leg lift	3 reps × 20–30 s (each side)
2C. Hamstrings	Level 1: Beginner	3–5 reps
	Level 2: Intermediate	7–10 reps
	Level 3: Advanced	12–15 reps
2D. Single-Leg Stance	Level 1: Hold the ball	2 reps × 30 s
	Level 2: Throwing ball with a partner	2 reps × 30 s
	Level 3: Test your partner	2 reps × 30 s
2E. Squats	Level 1: With toe raise	2 reps × 30 s
	Level 2: Walking lunges	2 reps × 30 s
	Level 3: One-leg squats (each leg)	2 reps × 30 s
2F. Jumping	Level 1: Vertical jump	2 reps × 30 s
	Level 2: Lateral jump	2 reps × 30 s
	Level 3: Box jump	2 reps × 30 s
**Part 3. Running Exercises (Approx. 2 min)**		
**Exercise**		**Repetitions**
Running across the pitch		2 reps
Running bounding		2 reps
Running plant and cut		2 reps

**Table 2:** Dynamic core stability training protocol for the DCS+FIFA 11+ group.

Additional Dynamic Core Exercises	Weeks 1–4 (Sets × Reps)	Weeks 5–8 (Sets × Reps)
Leg raises	3 × 12 reps	5 × 20 reps
Crunches	3 × 12 reps	5 × 20 reps
Superman	3 × 12 reps	5 × 20 reps
Plank hip twists	3 × 12 reps	5 × 20 reps
	Rest: 60 s between sets	Rest: 60 s between sets
Supine bridge	3 × 6 reps (10-s hold)	3 × 3 reps (10-s unilateral hold)
	Rest: 60 s between sets	Rest: 60 s between sets

**Table 3:** Baseline demographic and participant characteristics of the study groups.

Variable	Category	DCS+FIFA11+ (*n* = 20)	FIFA 11+ (*n* = 20)	Overall (*n* = 40)	*p*-Value
Age (years)		16.85 ± 2.78	16.50 ± 3.56	16.68 ± 3.16	0.72
Height (cm)		171.4 ± 5.61	168.9 ± 6.65	170.1 ± 6.20	0.21
Weight (kg)		55.90 ± 9.59	56.30 ± 8.96	56.10 ± 9.16	0.86
BMI (kg/m^2^)		18.87 ± 2.08	19.87 ± 2.66	19.37 ± 2.41	0.19
BMI Category	Underweight	5 (25%)	5 (25%)	10 (25%)	—
	Normal Weight	15 (75%)	13 (65%)	28 (70%)	
	Overweight	0	2 (10%)	2 (5%)	
Gender	Males	20 (100%)	20 (100%)	40 (100%)	
	Females	0 (0%)	0 (0%)	0 (0%)	
Dominant Foot	Right	15 (75%)	17 (85%)	32 (80%)	
	Left	5 (25%)	3 (15%)	8 (20%)	
Competitive Level	Amateur	20 (100%)	20 (100%)	40 (100%)	—

Values are reported as mean ± standard deviation (SD) for continuous variables and frequency (percentage) for categorical variables.

**Table 4:** Linear mixed model results for study outcomes.

Outcome	Group F (1,38)	Group *p*	Time F (2,76)	Time *p*	Group × Time F(2,76)	BH-Adjusted *p*	ηp^2^
DKV^o^ (R)	2.243	0.142	285.243	<0.001	0.565	0.913	0.015
DKV^o^ (L)	3.368	0.074	195.882	<0.001	2.874	0.130	0.070
Athletic Stability (R)	1.237	0.273	53.527	<0.001	2.836	0.130	0.069
Athletic Stability (L)	0.673	0.417	65.742	<0.001	12.169	<0.001	0.243
Hip Extensors (R)	0.021	0.885	72.286	<0.001	0.032	0.999	0.001
Hip Extensors (L)	0.010	0.921	135.047	<0.001	0.123	0.999	0.003
Hip Flexors (R)	0.296	0.590	39.605	<0.001	0.008	0.999	<0.001
Hip Flexors (L)	1.270	0.267	4.971	0.009	3.435	0.119	0.083
Hip Abductors (R)	0.225	0.638	78.105	<0.001	0.002	0.999	<0.001
Hip Abductors (L)	2.237	0.143	38.400	<0.001	5.423	0.025	0.125
Hip Adductors (R)	0.075	0.786	165.765	<0.001	2.173	0.215	0.054
Hip Adductors (L)	0.006	0.937	167.853	<0.001	3.238	0.119	0.079
Hip External Rotators (R)	3.098	0.086	60.306	<0.001	16.427	<0.001	0.302
Hip External Rotators (L)	1.669	0.204	168.450	<0.001	25.752	<0.001	0.404
Hip Internal Rotators (R)	0.040	0.842	164.627	<0.001	0.001	0.999	<0.001
Hip Internal Rotators (L)	0.394	0.534	179.240	<0.001	0.396	0.981	0.010

L: Left; R: Right.

**Table 5:** Estimated marginal means and 95% confidence intervals for the study outcomes.

Outcome	Group	Baseline EMM (95% CI)	4 Weeks EMM (95% CI)	8 Weeks EMM (95% CI)
DKV^o^ (R)	DCS+FIFA 11+	7.34 (6.72–7.97)	4.23 (3.61–4.86)	1.12 (0.50–1.75)
	FIFA 11+	7.60 (6.97–8.22)	4.75 (4.13–5.38)	1.91 (1.28–2.53)
DKV^o^ (L)	DCS+FIFA 11+	8.71 (8.04–9.37)	5.54 (4.86–6.21)	2.37 (1.69–3.04)
	FIFA 11+	8.67 (8.00–9.33)	6.18 (5.50–6.86)	3.70 (3.02–4.37)
Athletic Stability (R)	DCS+FIFA 11+	1.50 (1.24–1.75)	1.09 (0.83–1.34)	0.68 (0.43–0.94)
	FIFA 11+	1.53 (1.28–1.79)	1.28 (1.02–1.53)	1.02 (0.77–1.28)
Athletic Stability (L)	DCS+FIFA 11+	1.26 (1.05–1.46)	0.95 (0.74–1.15)	0.64 (0.44–0.84)
	FIFA 11+	1.19 (0.98–1.39)	1.06 (0.86–1.27)	0.94 (0.74–1.14)
Hip Extensors (R) (N)	DCS+FIFA 11+	248.7 (234.2–263.1)	254.7 (240.3–269.1)	260.7 (246.3–275.1)
	FIFA 11+	250.4 (236.0–264.8)	256.2 (241.8–270.6)	262.0 (247.5–276.4)
Hip Extensors (L) (N)	DCS+FIFA 11+	247.1 (232.1–262.2)	241.6 (226.6–256.6)	252.7 (237.7–267.7)
	FIFA 11+	248.2 (233.2–263.2)	243.0 (228.0–258.0)	253.4 (238.4–268.5)
Hip Flexors (R) (N)	DCS+FIFA 11+	281.5 (263.2–299.8)	287.8 (269.5–306.1)	294.1 (275.9–312.4)
	FIFA 11+	274.5 (256.3–292.8)	280.7 (262.4–299.0)	286.8 (268.6–305.1)
Hip Flexors (L) (N)	DCS+FIFA 11+	275.0 (262.0–288.1)	284.0 (270.9–297.1)	292.9 (279.9–306.0)
	FIFA 11+	273.3 (260.3–286.4)	274.2 (261.1–287.2)	275.0 (261.9–288.1)
Hip Abductors (R) (N)	DCS+FIFA 11+	214.6 (193.8–235.4)	220.0 (199.3–240.8)	225.5 (204.7–246.3)
	FIFA 11+	221.7 (201.0–242.5)	227.1 (206.4–247.9)	232.5 (211.8–253.3)
Hip Abductors (L) (N)	DCS+FIFA 11+	206.0 (189.9–222.2)	222.0 (205.8–238.1)	237.9 (221.7–254.1)
	FIFA 11+	197.9 (181.7–214.1)	205.1 (189.0–221.3)	212.3 (196.2–228.5)
Hip Adductors (R) (N)	DCS+FIFA 11+	215.3 (195.1–235.4)	220.7 (200.5–240.9)	226.2 (206.0–246.4)
	FIFA 11+	220.4 (200.2–240.5)	224.7 (204.5–244.9)	229.1 (208.9–249.2)
Hip Adductors (L) (N)	DCS+FIFA 11+	218.8 (199.7–238.0)	224.0 (204.8–243.1)	229.1 (210.0–248.2)
	FIFA 11+	219.0 (199.9–238.1)	222.9 (203.7–242.0)	226.7 (207.6–245.9)
Hip External Rotators (R) (N)	DCS+FIFA 11+	138.9 (129.4–148.3)	148.4 (139.0–157.8)	158.0 (148.5–167.4)
	FIFA 11+	133.7 (124.2–143.1)	136.7 (127.2–146.1)	139.7 (130.2–149.1)
Hip External Rotators (L) (N)	DCS+FIFA 11+	135.8 (125.4–146.3)	145.5 (135.1–155.9)	155.1 (144.7–165.6)
	FIFA 11+	131.6 (121.2–142.0)	135.8 (125.4–146.3)	140.0 (129.6–150.5)
Hip Internal Rotators (R) (N)	DCS+FIFA 11+	153.4 (139.7–167.0)	158.2 (144.5–171.8)	163.0 (149.3–176.6)
	FIFA 11+	151.4 (137.7–165.0)	156.2 (142.5–169.8)	161.0 (147.3–174.7)
Hip Internal Rotators (L) (N)	DCS+FIFA 11+	152.6 (139.9–165.3)	156.8 (144.1–169.4)	161.0 (148.3–173.6)
	FIFA 11+	147.2 (134.6–159.9)	151.0 (138.4–163.7)	154.8 (142.2–167.5)

Values are estimated marginal means from the linear mixed-effects models. DCS: Dynamic core stability; DKV^o^: Dynamic knee valgus angle; L: Left; R: Right; N: Newtons.

## Data Availability

The manuscript is a part of a PhD project. All relevant data supporting the findings of this study are included within the article. Additional datasets are available from the corresponding author upon reasonable request.

## Abbreviations

The following abbreviations are used in this manuscript:

BESS	Balance Error Scoring System
ACL	Anterior Cruciate Ligament
DCS+FIFA 11+	Dynamic Core Stability Training in addition to FIFA 11+
DKV	Dynamic Knee Valgus
FIFA	Fédération Internationale de Football Association
FUCP	Foundation University College of Physical Therapy
MD	Mean Difference
NWP	New Warm-Up Program
SD	Standard Deviation
